# Radiation Diagnostics of the Maxillofacial Region and Skeleton Bone Density in the Case of Vitamin D Insufficiency: A Pilot Study

**DOI:** 10.3390/life15030480

**Published:** 2025-03-17

**Authors:** Ekaterina Diachkova, Svetlana Tarasenko, Marina Skachkova, Yury Zhilkov, Natalia Serova, Anna Babkova, Beatrice Volel, Ekaterina Blinova, Elizaveta Kytko, Renata Meylanova, Victoria Zaborova, Olesya Kytko

**Affiliations:** 1 Department of Oral Surgery of Borovskiy Institute of Dentistry, Sechenov University, Mojaiskii val 11, 119048 Moscow, Russia; prof_tarasenko@rambler.ru (S.T.); marina.petukhova2014@ya.ru (M.S.); yuriyzhilkov2015@gmail.com (Y.Z.); 2 Department of Operative Surgery and Topographic Anatomy, I.M. Sechenov First Moscow State Medical University, 119435 Moscow, Russia; blinova_e_v@staff.sechenov.ru (E.B.); meylanova_r_d@staff.sechenov.ru (R.M.); kytko_o_v@staff.sechenov.ru (O.K.); 3 Department of Radiologic Diagnostics and Radiologic Therapy, Sechenov University, B.Pirogovskaya 6/2, 119992 Moscow, Russia; serova_n_s@staff.sechenov.ru (N.S.); babkova_a_a@staff.sechenov.ru (A.B.); 4 Sklifosovskyi Institute of Clinical Medicine, I.M. Sechenov First Moscow State Medical University, St. Trubetskaya, 8, Bld. 2, 119991 Moscow, Russia; volel_b_a@staff.sechenov.ru; 5 Department of Fundamental Medicine, MEPhI, 115409 Moscow, Russia; 6 Medical Prophylaxis Faculty, I.M. Sechenov First Moscow State Medical University, St. Trubetskaya, 8, Bld. 2, 119991 Moscow, Russia; kytkodoc@yandex.ru; 7 Department of Sport Medicine and Medical Rehabilitation, Sechenov University, 119435 Moscow, Russia; zaborova_v_a@staff.sechenov.ru; 8 Moscow Center for Advanced Studies, 123592 Moscow, Russia

**Keywords:** densitometry, cone beam computed tomography, DXA, QCT, vitamin D insufficiency, radiology, jawbone, skeleton

## Abstract

(1) Background: A decrease in bone mineral density has been noted not only in at-risk patients (e.g., postmenopausal women) but also in young and middle-aged individuals due to changes in lifestyle. The aim of the study was to find a possible correlation for dual-energy X-ray absorptiometry (DXA) and quantitative computed tomography (QCT) with cone beam computed tomography (CBCT) of the jaws. (2) Methods: A total of 24 patients (14 women and 10 men aged 25 to 50 years) with partial secondary tooth loss and vitamin D insufficiency underwent cone beam computed tomography of the jaws and skeletal mineral density assessment using DXA (*n* = 12) and QCT (*n* = 12). (3) Results: When conducting CBCT of the jaws, a predominance of bone tissue type D3 (350–850 Hu) on the upper jaw (*p* > 0.05 (F = 0.68) and D2 (850–1350 Hu) on the lower jaw (*p* > 0.05 (F = 1) was revealed. According to the results of QCT densitometry of the skeleton, signs of osteopenia were found in four patients (with vitamin D3 deficiency) (33%) according to DXA; signs of osteopenia were found in six patients (with severe deficiency and deficiency of vitamin D3) (50%). The difference between QCT and DXA was not significant (*p* > 0.05) for each group. The significant strong correlation between CBCT and DXA or QCT was not found (*p* > 0.05). (4) Conclusions: Primary changes in bone density can be detected earlier in the dental system using cone beam computed tomography of the jaws. At the same time, the question of using a specific densitometry method—DXA or QCT—remains open, as their results correlating with CBCT optical density was not approved.

## 1. Introduction

The main function of vitamin D is to regulate bone metabolism by maintaining intracellular and extracellular calcium and phosphorus homeostasis. Vitamin D deficiency contributes to decreased mineralization and increased bone resorption and consequently leads to the development of osteomalacia and bone loss. Vitamin D deficiency manifests itself in early childhood (4–12 months) with clinical and radiological symptoms of rickets and hypocalcemia. Alimentary rickets are a risk factor for fractures and can lead to tooth decay. In adulthood, vitamin D deficiency manifests itself as proximal muscle weakness and can also lead to osteomalacia and be a risk factor for osteoporosis [1].

There is a relationship between systemic osteoporosis and decreased jawbone mass, tooth loss, and risk factors for these conditions [2]. A positive correlation has been found between systemic bone loss and the degree of alveolar bone resorption [3]. Systemic osteoporosis affects the health of the dental system, since the bone tissue of the alveolar ridge, like the skeleton, is highly sensitive to the hormonal regulatory and control mechanisms of the body [4]. Postmenopausal osteoporosis and periodontal disease are also associated, and the loss of alveolar height and the number of lost teeth depends on the degree of osteopenia. It has been found that a decrease in skeletal bone mass correlates with a decrease in the height of the interdental bone septa, as well as a decrease in the attached gingiva [5]. This means that dentists need to work more closely with osteoporosis specialists, specifically endocrinologists, in the prevention, diagnosis, and treatment of this disease [6]. There is also a need for dental implantation to treat these patients and restore the functionality of their dental system.

Various densitometry methods are used to diagnose osteoporosis—methods for determining bone mineral density (BMD) with fracture risk assessment and body composition determination. Dual-energy X-ray absorptiometry (DXA) is the most common and accessible method of the radiation diagnostics of osteoporosis. It is the gold standard for diagnosing this disease in the absence of fractures [7]. Its advantage is low radiation exposure (0.03 mSv), which is important when using this method for screening [8]. However, a disadvantage of DXA is its limited ability to assess bone mineral density (BMD) in some patient groups, such as young patients or those with comorbidities such as type 2 diabetes. The latter are found to have normal or elevated mineralization levels, although bones are known to be more fragile [9]. During a DXA study, the projection bone mineral density (in g/cm^2^) is measured in the examined skeletal areas. After calculating the difference in absorption coefficients, three body components are calculated, namely body fat mass (BFM), mineral (bone) mass, and lean body mass (LBM). The main purpose of such a division is the high-precision measurement of bone mass [10]. Determination of the study area depends on the possibility of the reproducible measurement of projection bone density (g/cm^2^). The diagnosis of osteopenia or osteoporosis is based on a densitometric study in the Ward’s area and lumbar vertebrae (L1–L4) [10]. Degenerative changes in the vertebrae or an incorrectly calculated body mass index can provide erroneous results of BMD, which is also a disadvantage of this research method [11].

Quantitative bone computed tomography (QCT) is also a method of the radiation diagnostics of osteoporosis. It depends on the measurement parameters of trabecular and mineral bone density, geometry, and structure. Based on the measurements, a model is created based on the volumetric voxel architecture of the bone tissue structure. The three-dimensional measurement of BMD produced by QCT determines the amount of mineralized bone tissue per bone volume (g/cm^3^) [12]. This method is more effective because it displays the true volumetric density of bone tissue unlike the two-dimensional image of DXA, but it is more expensive [13]. However, the radiation dose when measuring QCT is much higher (1.5–2.9 mSv) [14].

Cone beam computed tomography (CBCT) creates images in three planes, which can be used to study a variety of parameters. Unlike DXA, CBCT is a more affordable procedure in terms of cost and also makes it easier for the doctor to interpret the data. In addition, the radiation dose is lower compared to all the methods listed above. Bone mineralization using CBCT can be assessed using a gray scale, while DXA creates one shade of gray [15]. Thus, DXA simplifies the complexity of bone, hiding important structural differences, which is a disadvantage of this examination method [13]. However, the disadvantage of this method of assessing bone density using shades of gray is the increased probability of error when scanning on different devices. This is due to different image settings, exposure, object position, the presence of artifacts in the bone, and different sensitivities of the devices [16].

Most studies that have examined bone mineral density have focused on postmenopausal patients. To our knowledge, there are no studies in the literature that have examined the relationship between all the above-mentioned scanning methods in young and middle-aged patients with tooth loss due to vitamin D deficiency. The aim of the study was to find a possible correlation for dual-energy X-ray absorptiometry (DXA) and quantitative computed tomography (QCT) with cone beam computed tomography (CBCT) of the jaws.

## 2. Materials and Methods

At the Department of Oral Surgery of the Institute of Dentistry of the I.M. Sechenov First Moscow State Medical University (Sechenov University) and the dental profile, 30 patients (16 women and 14 men) with a partial secondary absence of teeth with vitamin D insufficiency were treated with dental implantation from 2019 to 2024. The patients’ age ranged from 25 to 50 years, i.e., fell within the range of young and middle age according to the World Health Organization (WHO) classification (the average age was 35.2 ± 10.3 years). All patients were prescribed mandatory laboratory and instrumental examinations before the operation. Among the laboratory studies, special attention was paid to the level of liver enzymes, glycated hemoglobin, and thyroid and parathyroid hormones. The study was conducted in accordance with the Declaration of Helsinki and in agreement with the local ethics committee of Sechenov University (protocol no. 34-20 dated 9 December 2020).

Patients were stratified randomly in each age subgroup in equal numbers of men and women using a random number generator, where the numbers were coded for patients from the main randomized trial study (RCT, no. NCT04841213 on clinicaltrials.gov) and is a pilot project to test the potential presence of a correlation between changes in jawbone density and the skeleton based on radiological examination methods (CBCT, DXA, QCT).

This study is a pilot study, namely the first stage of the entire study, which helps in planning and modifying the main study. It is a smaller-scale study [17].

### 2.1. Inclusion Criteria

1. Availability of written informed consent from the patient to participate in the study.
2. Age not less than 18 and not more than 50 years.
3. Established diagnosis: tooth loss (K08.1-ICD 10), vitamin D3 imbalance (severe deficiency (<10 ng/mmol), and vitamin D3 deficiency (10–20 ng/mmol).
4. Absence of severe somatic pathology.
5. Endocrinologist approval for skeleton bone densitometry.

### 2.2. Exclusion Criteria of Main RCT

1. Age under 18 and over 50 years.
2. Pregnancy, breast feeding.
3. The presence of concomitant pathology: blood diseases, decompensated diabetes mellitus, immunodeficiency states, tuberculosis, or malignant neoplasms.

The number of patients is due to the minimum possible sample size for statistical analysis for both parametric and nonparametric methods for assessing the reliability of the study results, which was counted as 12 (alpha was 5%, power was 80%, sample size and 10—drop out) according to previous similar research [18]. According to sample size and inclusion criteria, 24 patients were included in two study groups. All patients had complaints; life history and disease history were collected, and patients underwent a clinical examination with an assessment of the dental system.

In addition to general radiography of the jaws and intraoral focal radiography, all patients underwent cone beam computed tomography of the jaws with a special emphasis on the area of the future operation using a PointNix 500 tomograph (PointNix, Seoul, Republic of Korea) when planning surgical intervention. The height and width of the alveolar ridge and the optical density of bone tissue in the area of the operation were determined.

CBCT was performed at the preoperative stage immediately after inserting dental implants—orthopantomography, during the insertion of the healing abutment—and orthopantomography or intraoral focal radiography were performed. For this purpose, the PointNix 500 tomograph (PointNix, Seoul, Republic of Korea) was used with the following characteristics: the slice thickness was 0.125–0.250 mm and the spatial resolution was 0.125 microns or higher.

To potentially determine changes in bone mineral density to test the hypothesis that D3 deficiency contributes to the development of osteopenia or osteoporosis signs not only in the maxillofacial area but also the skeleton, dual-energy X-ray absorptiometry (DXA) (*n* = 12) and quantitative bone computed tomography (QCT) (*n* = 12) were performed in randomized patients.

DXA was performed on a DEXXUM-3 X-ray bone densitometer (Osteosys, Seoul, Republic of Korea). The results were obtained as bone mineral density (BMD) in g/cm^2^ as a T-score—the ratio of the patient’s actual bone mass to the typical (maximum) bone mass of young healthy patients of the same sex, calculated as the standard deviation (SD) value—and as a Z-score—the ratio of the patient’s bone mass to the average-age bone mass of the reference group, calculated as the standard deviation value. The slice thickness was 0.5 mm. Tomography mode: continuous, 1 min 25 s–3 min 50 s. Radiation dose during the examination—below 10 m Rem per examination (distance 5 m) and below 1 m Rem (distance 5 m). In individuals under 50 years of age, BMD was assessed using the Z-score and was considered low at values ≤ −2.0.

QCT was performed on a 640-spiral Toshiba Aquilion ONE computed tomography scanner (Toshiba, Tokyo, Japan). The slice thickness was 0.5 mm. Tomography mode: spiral, without intravenous contrast. The effective dose during the study was 3 mSv. The control points were lumbar vertebrae 1–3 (L1, L2, L3) and the neck of the left femur, in which the average mineral density was assessed according to T- and Z-criteria.

### 2.3. Methodology for Conducting Computed Densitometry

- During the procedure, when the density of bone tissue of the femoral neck and spine is measured, the patient lies on a soft ‘table’ of the tomograph. The table moves inside the gantry of the tomograph, and X-rays pass through the patient’s body and hit the detector.
- The patient should lie as still as possible to avoid blurring the image. He or she may even be asked to hold his or her breath for a few seconds.
- The test usually lasts 5–10 min. During the examination, the person does not experience any discomfort.

All patients underwent a control X-ray examination immediately after the insertion of dental implants, during the insertion of healing abutment (after 3 months), and then once a year. The observation period for patients ranged from 1 to 10 years after dental implantation; the skeleton bone density investigation was performed each year in patients after endocrinologist prescription according to guidelines for osteoporosis/osteopenia.

### 2.4. Statistics Analysis

Descriptive statistics included the mean, median minimal, and maximum for qualitative criteria (age, optical bone density, T- and Z-criteria, vitamin D level, etc.). For comparison between groups, the Mann–Whitney test was used after distribution normality checking with the Shapiro–Wilk test inside of the groups. Before and after treatment, the Wilcoxon test was performed. For checking the possible single correlations between the optical bone density of the jaws and the criteria of skeleton densitometry, the Pearson test was performed. Fisher’s exact test was performed for odds ratios in small distributions. The difference and correlation were counted as significant with probabilities not less than 95% (*p* < *0*.05). For statistical analysis, the program RStudio was used (version 4.2.2 (31 October 2022)).

## 3. Results

### 3.1. Jaws CBCT Results

When conducting CBCT of the jaws of the patients in this study and assessing the optical density of the area of future dental implantation, the prevalence of bone tissue type D3 (350–850 Hu) on the upper jaw (six patients—50%) and D2 (850–1350 Hu) on the lower jaw (six patients—50%) was revealed in two cases with a long-term absence of teeth (at least 1 year)—with type D1 (>1350 Hu) on the lower jaw (16.7%) and in one case for the upper jaw (8.7%) (Table 1).

All patients underwent CBCT of the jaw around the inserted dental implants at 3 months. The types of jawbone structure with density in the area of tooth loss are presented in Table 2.

**Figure 1:**
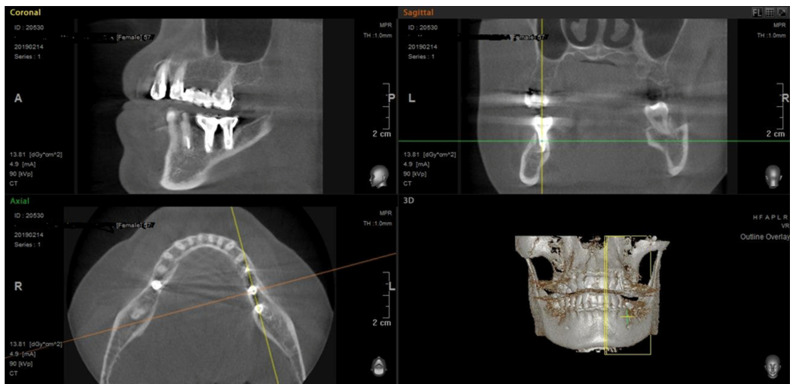
Patient’s CBCT example 6 years after operation. The D2-type bone density around dental implants has been shown in the presence of both types of bone (cortical and sponge) and the absence of pathological bone resorption signs (zone of interest is marked with cross of yellow and red lines).

During a control X-ray examination after the insertion of dental implants, no marginal or apical bone resorption was detected in the area of the installed dental implants (Figure 1).

### 3.2. Bone Tissue Mineral Density Evaluation Results

#### 3.2.1. Skeleton QCT Densitometry

**Figure 2:**
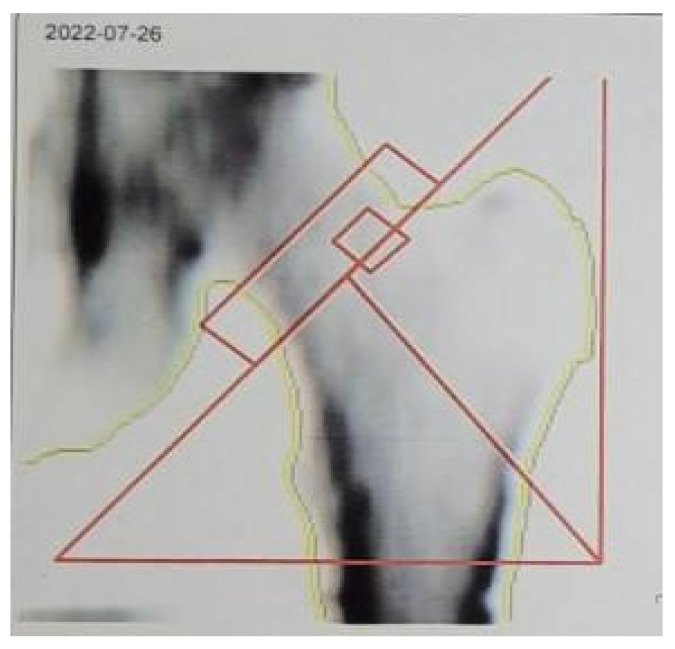
Patient’s QCT example. Zone of scan and lines for bone mineral density assessment in the area of the left thigh (zone of interest is marked with red lines).

Twelve patients in the study had severe deficiency (<10 ng/mmol) and vitamin D3 deficiency (10–20 ng/mmol). They underwent skeleton QCT densitometry. The study revealed signs of osteopenia in four patients (33.3%) (with vitamin D3 deficiency) out of twelve (Figure 2). The results of the study are presented in Table 3.

#### 3.2.2. DXA Results

**Figure 3:**
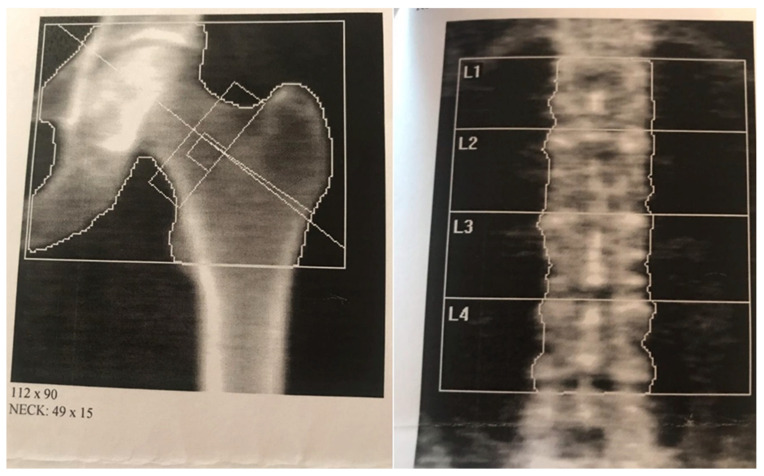
Patient’s DXA results example, with reference areas for mineral bone density assessment—the femur neck (**left figure**) and lumbar vertebrae 1st–4th (**right figure**).

Skeletal DXA was performed in 12 patients with severe deficiency (<10 ng/mmol) and vitamin D deficiency (10–20 ng/mmol). The results of the study revealed signs of osteopenia in six patients (with severe deficiency and vitamin D3 deficiency) (50%) out of twelve (Figure 3). The study results are presented in Table 4.

### 3.3. Comparison of DXA and QCT and Their Correlation with Jawbone Optical Density According to CBCT

The comparison of main criteria according to DXA and QCT was performed, and the results are presented in Table 5.

It should be said that for true comparison, the accuracy of methods must be assessed, which is impossible on a small sample size and for the pilot study itself.

The results of possible correlation of jawbone optical density according to CBCT before treatment and skeleton bone mineral density according DXA/QCT are introduced in Table 6.

For jawbone optical bone density on CBCT and skeletal mineral bone density assessment with DXA or QCT, there was no strong significant correlation.

Thus, radiation methods of bone tissue diagnostics—both the dental system and the skeleton with a pronounced deficiency and deficiency of vitamin D3—can demonstrate a decrease in optical density (with CBCT of the jaws) and mineral density of bone tissue (with CBCT and DXA of the skeleton), which requires a full-fledged randomized clinical study in relation to the latter indicator.

## 4. Discussion

Vitamin D3 deficiency is increasingly recorded worldwide every year, regardless of the country’s location [19]. There are a significant number of reasons for the occurrence of deficiency of this vitamin. However, a few factors were identified that explained the differences among population groups. One of them is the difference in the degree of exposure to sunlight, as well as its duration and intensity [20]. A special criterion for the success of the synthesis is the angle of incidence of the sun’s rays, since if there is a lack of it, the process is not activated [21]. Another reason was the characteristics of the person: age and the presence of concomitant diseases that can reduce the body’s ability to absorb vitamin D3 [22]. In addition, the ability to synthesize vitamin D3 in the body is affected by skin color and the use of sunscreen, which slows down this process [20]. Thus, a deficiency condition can be observed in many patients. It is necessary to take this into account when planning dental intervention and to preliminarily study the condition of the bone in the implantation area.

The most complete information on the state of the BMD can be obtained using DXA and QCT. As mentioned earlier, the advantage of the DXA method is the low radiation exposure. Its disadvantage is that the study results are affected by dense formations located at the edges of the vertebral bodies, as well as paravertebral soft tissues, the size of the bone being examined, and the patient’s position. In turn, the QCT method allows for a more accurate determination of BMD in some cases (with pronounced changes in the bone and joint system) than DXA and allows for the use of spine and femur scans for analysis. Both methods allow for the provision of data in the T- and Z-criteria format. The T-criterion is characterized as the standard deviation above or below the peak bone mass indicator in young women aged 20–29 years. The recommended reference interval was obtained from the database of the Third National Health and Nutrition Examination Survey [18]. The International Society for Clinical Densitometry (ISCD) recommends using the ethnically and racially adjusted Z-score instead of the T-score. It is a standard deviation from the meaning, with values of −2.0 and below being interpreted as ‘low BMD for chronological age’ or ‘below age-appropriate values’ and values above −2.0 being ‘within age-appropriate values’. The following classification proposed by the WHO is used to assess BMD status: in postmenopausal women, the diagnosis of osteoporosis corresponds to T-score values of −2.5, the diagnosis of osteopenia corresponds to values of −1.0 to −2.5, and the normal value is −1.0 and above [23]. For premenopausal healthy women, the WHO classification is not applicable; therefore, the Z-score is used to interpret DXA densitometry results, and secondary causes of low BMD are usually required to diagnose osteoporosis. The WHO classification is not fully applicable to interpreting DXA values in men. Thus, in men under 50 years of age, it is not recommended to diagnose osteoporosis based only on densitometric results. This nuance is a disadvantage of the DXA method and creates difficulties for correct diagnosis. As a result, there is a higher radiation load for the patient, as well as financial losses, which can create a difficult situation. In men aged 50 to 65 years, osteoporosis is detected with a T-score below −2.5 and the presence of other risk factors for fracture development. In men over 65 years of age, osteoporosis is diagnosed with a T-score of −2.5 or lower. According to the decision of the ISCD position committee, the BMD classification proposed by the WHO is not applicable to children.

QCT allows for separate scanning of spongy and compact bone tissue. However, this method of examination is accompanied by the highest radiation load than the other methods we report on. This procedure is more expensive not only for the patient but also for the clinic. The cost of this device is initially higher. At the same time, the need for monthly calibration with a special phantom is noted, which is why this method is more expensive compared to DXA.

Often, in the presence of diseases that cause metabolic disorders, periodontal tissue pathologies develop, resulting in a secondary loss of teeth [24,25]. For this reason, the treatment of patients with vitamin D deficiency using prosthetics supported by dental implants may be limited due to the risk of their rejection. Since timely and correct treatment increases the chance of successful implant engraftment in the future, there is a need for additional diagnostic methods for patients as part of their preparation for surgery, including DXA and QCT methods [26]. Unfortunately, the possibility of using only one technique is not feasible, since these methods are not able to replace each other. Despite this, there are studies that try to reflect the possibility of comparability with these two methods. However, difficulty arises in the simultaneous calibration of the mass of minerals and their spatial distribution because DXA is characterized by fan beam geometry, while QCT is parallel and has other algorithmic details. Thus, the study by B. C. Khoo showed the possibility of calibration through complex calculations that allowed for the elimination of numerical differences between the corresponding structural geometric measurements. However, the authors note that this cross-calibration may not work when changing the group sample and other data [27].

It is well known that bone constantly undergoes synthesis and resorption processes regulated by local and general factors. In the jawbones, these processes occur much faster than in the bones of other parts of the skeleton. Obviously, general vitamin D insufficiency will also affect the bones of the facial part of the skull. The features and causes of a decrease in BMD in the maxillofacial region are mentioned in various scientific papers about DXA and QCT. Thus, NM. Elkersh et al. compared DXA and CBCT data of the maxillofacial region in postmenopausal women with suspected osteoporosis [28]. A positive correlation was noted between these methods. According to DXA, BMD was reduced in spine or femur, and according to CBCT, this reduction was noted in the mandible. Based on this, the authors concluded that screening diagnostics of postmenopausal women for osteoporosis is necessary at the stage of radiological examination during the initial visit to the dentist if they are planning treatment with dental implants [29]. We observed a similar fact in patients who underwent DXA or QCT, so we fully agree with the authors on the importance of additional diagnostics. Primary changes in bone structure can be detected more quickly in the alveolar process area, which is diagnosed using CBCT. Thus, it is possible to diagnose mineral metabolism disorders in time and correct them together with an endocrinologist, thereby preventing the risk of possible complications during dental intervention. MG Sghaireen et al. conducted a study in which they compared the relationship between DXA and CBCT results in the upper and lower jaws when planning dental implantation. The study involved 81 patients, including those with and without diagnosed osteoporosis. DXA values were displayed in points corresponding to the T-criterion, with CBCT values in grayscale. A correlation was noted between the average T-criterion values and CBCT results in different parts of the jaws. The correlation was maintained even when the indicators decreased to values corresponding to osteoporosis. The results of this study confirm the feasibility of using CBCT scans obtained at the stage of dental implantation planning as a screening tool for jaw changes such as osteopenia and osteoporosis [30]. This paper highlights the importance of vitamin D supplementation for restoring bone mineral density, which is comparable to the treatment of patients in our study. A correlation between gray values between CBCT and DXA exists and is supported by several sources [15,31,32]. We also observed this parameter in our work when comparing CBCT results with DXA and QCT. However, it is important to note that the correlation between CBCT results and skeletal densitometry does not confirm cause-and-effect relationships. In addition, it is equally important that CBCT may have variability in Hounsfield (HU) values, which arises from the use of different CT scanning protocols [33]. That is why it is important for the correctness of the studies to use the same scanning protocol. It consists of the fact that CBCT is carried out on the same device without changing the parameters and settings.

O.O. Yanushevich and M.V. Kozlova conducted a study on the impact of osteoporosis on the results of dental implantation at the A.I. Evdokimov Moscow State Medical University. The study involved 25 women and 25 men aged 54 to 65. Osteoporosis was detected in 40 patients according to DXA data. Depending on the drug therapy with bisphosphonates, the patients were divided into several groups. As a result of repeated radiological studies, it was found that the use of bisphosphonates together with vitamin D3-forte and calcium preparations for 3–6 months in the prescribed doses contributes to an increase in mineral metabolism indices, making further dental implantation possible in patients with osteoporosis [30]. There is information on the use of densitometry in patients with periodontitis and concomitant rheumatoid arthritis (RA). S.V. Tarasenko and A.A. Makarevich report that patients with RA had a reduced T-criterion value relative to healthy patients from the control group [34]. A decrease in densitometry values was also noted with the progression of periodontitis from I to III severity. In 2015, L. Yu. Ostrovskaya studied the treatment of chronic generalized periodontitis in postmenopausal women. In this study, BMD was assessed using DXA in postmenopausal women with and without a diagnosis of osteoporosis. The study results showed that chronic generalized periodontitis (CGP) in systemic osteoporosis, even under conditions of proper oral hygiene, was more aggressive and generalized, unlike patients from the control group, whose BMD was within normal values. At the same time, the severity of CGP in patients from both groups did not differ significantly. Thus, a conclusion was drawn about the effect of a decrease in skeletal BMD on periodontal bone density and the development of non-inflammatory periodontitis [35]. Also, M. A. Ufimtseva and co-authors used DXA to analyze the BMD of patients with osteoporosis caused by the long-term use of glucocorticosteroids against the background of acantholytic pemphigus (AP) with manifestations in the oral cavity. Twenty-six patients with bullous dermatosis were examined, including 12 patients with AP, including those with erosions localized on the oral mucosa. Among the examined, four people with AP (33.3%) were diagnosed with glucocorticoid osteoporosis, and another four (33.3%) showed signs of osteopenia [36]. Thus, the above analysis of available sources showed frequent changes in BMD by the type of osteopenia and osteoporosis among dental patients. However, there is a lack of studies devoted to the comparison of the effectiveness of DXA and QCT with sufficient duration, especially in dental practice. The limitations of our study include the small number of patients and the inability to compare the results of DXA and QCT of the skeleton due to the inadmissibility of recalculating the coefficients in the studies.

### Limitations of Our Study

The limitations of our study include the small number of patients, the inability to compare the results of the skeletal bone densitometry method accuracy, and to transfer the results of the study to each patient due to absence of the possibility to perform both DXA and QCT of the skeleton (radiological overload). Also, due to the small number of study participants, we did not identify and analyze the results depending on the age and gender of the patients.

## 5. Conclusions

Radiation methods of bone tissue diagnostics with pronounced deficiency and a deficiency of vitamin D3 can demonstrate a decrease in optical density (with CBCT of the jaws) and mineral density of bone tissue (with CBCT and DXA of the skeleton) that could possibly affect the results of dental implantation, but randomized studies are required regarding the latter indicator. In addition, primary changes in bone density can be detected earlier in the dental system using cone beam computed tomography of the jaws. At the same time, the question of using a specific densitometry method—DXA or QCT—remains open, as their results correlating with CBCT optical density was not approved.

## Figures and Tables

**Table 1:** Jawbone density types in the area of tooth loss at the stage of dental implantation planning with CBCT (*n* = 24).

Jawbone Density Type	Maxilla (*n* = 12)	Mandible (*n* = 12)	*p* (Fisher’s Exact Test)
D1 (>1350 Hu)	1 (8.4%)	2 (16.7%)	>0.05 (F = 1)
D2 (850–1350 Hu)	5 (41.6%)	6 (50%)	>0.05 (F = 1)
D3 (350–850 Hu)	6 (50%)	4 (33.3%)	>0.05 (F = 0.68)
D4 (<350 Hu)	0	0	NA *

* NA—not available.

**Table 2:** Jawbone density types in the area of tooth loss 3 months after dental implantation with CBCT (*n* = 24).

Jawbone Density Type	Maxilla (*n* = 12)	Mandible (*n* = 12)	*p* (Fisher’s Exact Test)
D1 (>1350 Hu)	1 (8.4%)	2 (16.7%)	>0.05 (F = 1)
D2 (850–1350 Hu)	11	10	>0.05 (F = 1)
D3 (350–850 Hu)	0	0	NA *
D4 (<350 Hu)	0	0	NA *

* NA—not available.

**Table 3:** QCT densitometry results in patients with teeth loss and vitamin D deficiency (*n* = 12).

Region	Me ± m Median Min–Max
Vertebral bone mineral density (L1, L2, L3) (Mean mineral density mg/cm^3^)	149.7 ± 39.3 153.6 101.1–198.9
T-criterion of left femur neck	0.05 ± 0.71 0.46 −0.95–0.64
Z-criterion of left femur neck	0.42 ± 0.97 0.7 −0.88–1.46

**Table 4:** DXA results in randomly chosen patients (*n* = 12).

Region	Me ± m Median Min–Max
Vertebral bone mineral density (L1, L2, L3) Mean mineral density mg/cm^3^	108.3 ± 5.0 110.6 101.2–113.2
T-criterion of left femur neck	0 ± 1.4 0 −1.5–1.5
Z-criterion of left femur neck	0.12 ± 0.1 0.15 0.1–0.2

**Table 5:** Comparison of DXA and QCT among patients with vitamin D deficiency (*n* = 24).

Criterion	Group 1 QCT (*n* = 12)	Group 2 DXA (*n* = 12)
Vertebral bone mineral density (L1, L2, L3) (mg/cm^3^)	*p* (Mann–Whitney test) (0.068) > 0.05	
T-criterion of left femur neck	*p* (Mann–Whitney test) (1) > 0.05	
Z-criterion of left femur neck	*p* (Mann–Whitney test) (0.17) > 0.05	

**Table 6:** Correlation between CBCT of the jaws and skeletal mineral bone density on DXA/QCT (*n* = 24).

Method of Bone Density Assessment	Skeletal QCT (*n* = 12)	Skeletal DXA (*n* = 12)
CBCT of jaws (*n* = 24)	Pearson Correlation Coefficient R = −0.16 *p* > 0.05	Pearson Correlation Coefficient R = 0.15 *p* > 0.05

## Data Availability

Data are available upon request due to restrictions.

## Abbreviations

The following abbreviations are used in this manuscript:

BMD	Bone mineral density
CBCT	Cone beam computed tomography
DI	Dental implantation
DXA	Dual-energy X-ray absorptiometry
QCT	Quantitative computed tomography

## References

[1] Pigarova E.A., Glazieva V.S., Povaliaeva A.A., Dzeranova L.K., Belovalova I.M., Dedov I.I. (2024). Features of diagnosis and treatment of patients with vitamin d deficiency in real clinical practice. Obes. Metab..

[2] Wang C.J., McCauley L.K. (2016). Osteoporosis and Periodontitis. Curr. Osteoporos. Rep..

[3] Giro G., Chambrone L., Goldstein A., Rodrigues J.A., Zenóbio E., Feres M., Figueiredo L.C., Cassoni A., Shibli J.A. (2015). Impact of osteoporosis in dental implants: A systematic review. World J. Orthop..

[4] NIH Consensus Development Panel on Osteoporosis Prevention, Diagnosis, and Therapy (2001). Osteoporosis prevention, diagnosis, and therapy. JAMA.

[5] Wactawski-Wende J. (2001). Periodontal diseases and osteoporosis: Association and mechanisms. Ann. Periodontol..

[6] Dodd D.Z., Rowe D.J. (2014). The relationship between postmenopausal osteoporosis and periodontal disease. Am. Dent. Hyg. Assoc..

[7] Blake G.M., Fogelman I. (2009). The clinical role of dual energy X-ray absorptiometry. Eur. J. Radiol..

[8] Andreoli A., Scalzo G., Masala S., Tarantino U., Guglielmi G. (2009). Body composition assessment by dual-energy X-ray absorptiometry (DXA). La Radiol. Medica.

[9] Napoli N., Strollo R., Paladini A., Briganti S.I., Pozzilli P., Epstein S. (2014). The alliance of mesenchymal stem cells, bone, and diabetes. Int. J. Endocrinol..

[10] Nizovtsova L.A., Morozov S.P., Petryaykin A.V., Bosin V.Y., Sergunova K.A., Vladzimirskiy A.V., Shantarevich M.Y. (2018). On the unification of bone densitometry and interpretation of its results. J. Radiol. Nucl. Med..

[11] Setiawati R., Di Chio F., Rahardjo P., Nasuto M., Dimpudus F.J., Guglielmi G. (2016). Quantitative assessment of abdominal aortic calcifications using lateral lumbar radiograph, dual-energy X-ray absorptiometry, and quantitative computed tomography of the spine. J. Clin. Densitom..

[12] Villarraga-Gómez H., Herazo E.L., Smith S.T. (2019). X-ray computed tomography: From medical imaging to dimensional metrology. Precis. Eng..

[13] Amstrup A.K., Jakobsen NF B., Moser E., Sikjaer T., Mosekilde L., Rejnmark L. (2015). Association between bone indices assessed by DXA, HR-pQCT and QCT scans in post-menopausal women. J. Bone Miner. Metab..

[14] Koch V., Hokamp N.G., Albrecht M.H., Gruenewald L.D., Yel I., Borggrefe J., Wesarg S., Eichler K., Burck I., Gruber-Rouh T. (2021). Accuracy and precision of volumetric bone mineral density assessment using dual-source dual-energy versus quantitative CT: A phantom study. Europ. Radiol. Experim..

[15] Marquezan M., Lau T.C., Mattos C.T., da Cunha A.C., Nojima L.I., Sant’Anna E.F., de Souza M.M.G., de Souza Araújo M.T. (2012). Bone mineral density: Methods of measurement and its influence on primary stability of miniscrews. Angle Orthod..

[16] Shokri A., Ramezani L., Bidgoli M., Akbarzadeh M., Ghazikhanlu-Sani K., Fallahi-Sichani H. (2018). Effect of field-of-view size on gray values derived from cone-beam computed tomography compared with the Hounsfield unit values from multidetector computed tomography scans. Imaging Sci. Dent..

[17] In J. (2017). Introduction of a pilot study. Korean J. Anesthesiol..

[18] Boehm E., Kraft E., Biebl J.T., Wegener B., Stahl R., Feist-Pagenstert I. (2024). Quantitative computed tomography has higher sensitivity detecting critical bone mineral density compared to dual-energy X-ray absorptiometry in postmenopausal women and elderly men with osteoporotic fractures: A real-life study. Arch. Orthop. Trauma Surg..

[19] Siddiqee M.H., Bhattacharjee B., Siddiqi U.R., MeshbahurRahman M. (2021). High prevalence of vitamin D deficiency among the South Asian adults: A systematic review and meta-analysis. BMC Public Health.

[20] Rathish N., Maseeh A. (2012). Vitamin D: The “sunshine” vitamin. J. Pharmacol. Pharmacother..

[21] Yeum K.-J., Song B.C., Joo N.-S. (2016). Impact of Geographic Location on Vitamin D Status and Bone Mineral Density. Int. J. Environ. Res. Public Health.

[22] Mostafa W.Z., Hegazy R.A. (2015). Vitamin D and the skin: Focus on a complex relationship: A review. J. Adv. Res..

[23] Dimai H.P. (2017). Use of dual-energy X-ray absorptiometry (DXA) for diagnosis and fracture risk assessment; WHO-criteria, T-and Z-score, and reference databases. Bone.

[24] Mongkornkarn S., Suthasinekul R., Sritara C., Lertpimonchai A., Tamsailom S., Udomsak A. (2019). Significant association between skeletal bone mineral density and moderate to severe periodontitis in fair oral hygiene individuals. J. Investig. Clin. Dent..

[25] Bartl R., Bartl C. (2019). Oral bone loss due to periodontitis and systemic osteoporosis. Osteoporos. Man. Prev. Diagn. Manag..

[26] Aghaloo T., Pi-Anfruns J., Moshaverinia A., Slim D., Grogan T., Hadaya D. (2019). The Effects of Systemic Diseases and Medications on Implant Osseointegration: A Systematic Review. Int. J. Oral Maxillofac. Implant..

[27] Khoo BC C., Brown K., Zhu K., Price R.I., Prince R.L. (2014). Effects of the Assessment of 4 Determinants of Structural Geometry on QCT- and DXA-Derived Hip Structural Analysis Measurements in Elderly Women. J. Clin. Densitom..

[28] Elkersh N.M., Talaab M.R., Ahmed W.M., Gaweesh Y.S. (2019). Utility of cone beam computed tomography of the mandible in detection of osteoporosis in postmenopausal women. Alex. Dent. J..

[29] Sghaireen M.G., Ganji K.K., Alam M.K., Srivastava K.C., Shrivastava D., Ab Rahman S., Patil S.R., Al Habib S. (2020). Comparing the Diagnostic Accuracy of CBCT Grayscale Values with DXA Values for the Detection of Osteoporosis. Appl. Sci..

[30] Yanushevich O.O., Kozlova M.V., Mkrtumyan A.M., Belyakova A.S., Kozlova L.S. (2014). The characteristics of dental implantation in patients with osteoporosis. Treat. Profilaxys.

[31] Shokri A., Ghanbari M., Maleki F.H., Ramezani L., Amini P., Tapak L. (2019). Relationship of Gray Values in Cone Beam Computed Tomography and Bone Mineral Density Obtained by Dual Energy X-ray Absorptiometry. Oral Surg. Oral Med. Oral Pathol. Oral Radiol..

[32] Barngkgei I., Al Haffar I., Khattab R. (2014). Osteoporosis prediction from the mandible using cone-beam computed tomography. Imaging Sci. Dent..

[33] Zurl B., Tiefling R., Winkler P., Kindl P., Kapp K.S. (2014). Hounsfield units variations: Impact on CT-density based conversion tables and their effects on dose distribution. Strahlenther. Onkol..

[34] Tarasenko S.V., Dydykina I.S., Nikolaeva E.N., Tsarev V.N., Makarevich A.A. (2019). The importance of additional methods for examining patients with chronic generalized periodontitis in combination with rheumatoid arthritis. Clin. Stomatol..

[35] Ostrovskaya L.U., Khanina A.I. (2015). Treatment of chronic generalized periodontitis at women in the period of postmenopause. Saratov J. Med. Sci. Res..

[36] Ufimtseva M.A., Bochkarev Y.M., Gurkovskaya E.P., Puhtinskaya P.S., Nikolaeva K.I., Lesnaya O.D. (2016). Osteoporosis as a result of the long-term administration of glucocorticoids in patients suffering from acantholytic pemphigus. Vestn. Dermatol. I Venerol..

